# Molecular Features of Wheat Endosperm Arabinoxylan Inclusion in Functional Bread 

**DOI:** 10.3390/foods2020225

**Published:** 2013-06-17

**Authors:** Weili Li, Hui Hu, Qi Wang, Charles J. Brennan

**Affiliations:** 1 Department of Food and Tourism Management, Manchester Metropolitan University, Manchester, M14 6HR, UK; E-Mails: w.li@mmu.ac.uk (W.L.); qiqitutu2781@yahoo.com (H.H.); 2 Agriculture and Agri-Food Canada, Guelph Food Research Centre, Guelph N1G 5C9, Canada; E-Mail: qi.wang@agr.gc.ca; 3 Department of Wine, Food and Molecular Biosciences, Lincoln University, Lincoln 7647, New Zealand

**Keywords:** arabinoxylans, endoxylanase, functional bread, extractability, molecular weight

## Abstract

Arabinoxylan (AX) is a major dietary fibre component found in a variety of cereals. Numerous health benefits of arabinoxylans have been reported to be associated with their solubility and molecular features. The current study reports the development of a functional bread using a combination of AX-enriched material (AEM) and optimal commercial endoxylanase. The total AX content of bread was increased to 8.2 g per 100 g available carbohydrates. The extractability of AX in breads with and without endoxylanase was determined. The results demonstrate that water-extractable AX (WE-AX) increased progressively through the bread making process. The application of endoxylanase also increased WE-AX content. The presence of 360 ppm of endoxylanase had positive effects on the bread characteristics in terms of bread volume and firmness by converting the water unextractable (WU)-AX to WE-AX. In addition, the molecular weight (Mw) distribution of the WE-AX of bread with and without endoxylanase was characterized by size-exclusion chromatography. The results show that as the portion of WE-AX increased, the amount of high Mw WE-AX (higher than 100 kDa) decreased, whereas the amount of low Mw WE-AX (lower than 100 kDa) increased from 33.2% to 44.2% through the baking process. The low Mw WE-AX further increased to 75.5% with the application of the optimal endoxylanase (360 ppm).

## 1. Introduction

Arabinoxylan (AX) is a major dietary fibre component found in a variety of cereals [1,2,3,4,5,6]. Recent research has focused on the bioactivities and health benefits of cereal AXs [6,7]. Based on the scientific data obtained in intervention studies [8,9], the European Food Safety Authority (EFSA) has approved that the health benefit of the reduction of post-prandial glycaemic response can be claimed on foods containing more than 4.8 g of wheat endosperm AXs per 100 g available carbohydrates. In addition, the studies on the immune stimulation properties of wheat bran AXs have demonstrated that the AXs with an average molecular weight (Mw) of 32.52 kDa extracted with endoxylanase showed a stronger activity than those with an average Mw of 351.7 kDa extracted with alkali solution [10,11]. Thus, the immune stimulation properties of extracted wheat bran AXs seem to be related with their molecular features. It is also interesting to observe from recent studies that as wheat bran AXs were depolymerised to oligosaccharide (AXOS), they showed a stronger prebiotic activity [5,6,7,12,13,14,15]. Based on these scientific reports, wheat bran extract, AXOS, has been approved as a prebiotic for foods by the American Food Drug Administration [16]. 

Thus, the incorporation of bioactive AXs into foods becomes an alternative method to enhance the nutritional values of foods. The aims of this study are to develop a functional bread with the health benefits and to examine the changes in the extractability and molecular features of arabinoxylans during bread processing. The results should provide a greater understanding of the potential for nutritional enhancement of breads using the prebiotic and immune stimulating activities of AXs. 

## 2. Materials and Methods

### 2.1. Materials

AXs-enriched material (AEM) was kindly provided by Henan Lianhua Monosodium Glutamate Group Co. Ltd. (Xiangchen, China). Endoxylanase (Pentopan Mono BG) was kindly supplied by Novozyme, Bagsvaerd, Denmark. UK bread flour (white flour) was purchased from a UK milling company, Smith Mills (Worksop, UK) with 12.1% protein, 14% water and 58% water absorption.

### 2.2. Chemicals

d-(+)-Xylose, purchased from Acros Organics, Loughborough, UK, was used to prepare the standards for determining the quantity of the AXs for the samples. Eight pullulan (with no side chain) standards purchased from Shodex (Shanghai, China) of varying molecular weights (ranging from 5000–800,000) were used for SEC-HPLC to characterise the Mw of AXs. NaNO_3_ and NaN_3_ for the HPLC mobile phase were purchased from Sigma-Aldrich, Gillingham, UK. 

Termamyl (α-amylase), type XII-A, *Bacillus licheniformis* A3403-1MU and pancreatin, porcine pancreas, P7545-100G, were purchased from Sigma-Aldrich Co, Brøndby, Denmark. Pepsin, porcine gastric mucosa, EC-3.4.23.1, was purchased from Merck, Gernsheim, Germany. Those enzymes were used in the purification of AX from AEM and bread samples.

### 2.3. Methods

#### 2.3.1. Bread Making

Breads were prepared from the formulation, as shown in Table 1, with and without AEM and in the presence and absence of endoxylanase.

**Table 1:** Bread recipe. AEM, arabinoxylan (AX)-enriched material.

	White bread (g)	Bread + AEM (g)	Bread + AEM + 360 ppm endoxylanase (g)	Bread + AEM + 600 ppm endoxylanase (g)
Strong flour	600	408	408	408
Salt	8.6	8.6	8.6	8.6
Sugar	6	6	6	6
Fat	6.6	6.6	6.6	6.6
Compact yeast	37.3	37.3	37.3	37.3
Bread improver	6	6	6	6
Arabinoxylan enriched material	0	192	192	192
Water	336	390	390	390
Endoxylanase	0	0	0.036	0.06

#### 2.3.2. Bread Firmness Determination

The firmness of bread was determined by the use of a texture analyzer of TA.XT, following the AACC (74-09) standard method [17].

#### 2.3.3. Water-Extractable AX (WE-AX) Preparation

Fresh bread samples were milled in a coffee blender for 30 s. Bread samples (50 g) were extracted with 200 mL water with constant stirring for 60 min at room temperature and then centrifuged (6000× *g*, 30 min). The supernatant (50 mL) was transferred to an Erlenmeyer flask, and 0.1 M phosphate buffer was added, until pH 6.0 was attained. The sample was then vortexed to create a suspension, and 5 mL termamyl (α-amylase) was added and incubated in a boiling water bath for 20 min. The sample was then cooled to room temperature, and the pH of the solution adjusted to 1.5 with 4 M HCl. Protein degradation was achieved with the addition of 5 g pepsin and incubation at 40 °C in a shaking water bath for 60 min. Subsequently, the sample’s pH was adjusted to 6.8 with 4 M NaOH, and 5 g pancreatin was added prior to incubation at 40 °C for 60 min. The sample was cooled to room temperature and centrifuged (6000× *g*, 30 min). The water-extractable AX (WE-AX) was then precipitated from the supernatant by stepwise addition of absolute ethanol to a final concentration of 65% (v/v). The mixture was stirred for 30 min and kept at 4 °C overnight. The precipitate was separated by centrifugation (6000× *g*; 30 min; 4 °C) and dissolved in distilled water (1 L), and then ethanol was added to a final concentration of 60% (v/v) and was stirred as above and kept overnight at 4 °C. The precipitate was recovered by centrifugation (6000× *g*; 30 min; 4 °C) and washed twice with 96% (v/v) ethanol (500 mL).The acetone (500 mL) was added and was stirred for 120 min, then centrifuged at 6000× *g*, at 4 °C for 30 min. The final precipitate (WE-AX) was dried for 24 h at 45 °C in a drying oven.

#### 2.3.4. Determination of AX Content

The AX content of the AEM and the WE-AX and water unextractable AX (WU-AX) fractions of the tested breads was determined using the method described by Douglas [18]. A standard curve of xylose was constructed for determination of the xylose content of the AEM and bread samples, which, in turn, was used to calculate the percentage of AX in the extracts based on an average A/X ratio of 0.6 as tested in mono-sugar composition analysis (the results are not shown in this paper). 

#### 2.3.5. Characterization of Molecule Weights of AXs

A size exclusion high-pressure liquid chromatography system, Shimadzu LC-10 (Shimdzu Corporation, Kyoto, Japan), with JASCO RI-2031 Refractive Index (RI) Detector (Jasco Corporation, Tokyo, Japan) and BioSep-SEC-S 4000 and BioSep-SEC-S 3000 columns (Phenomenex, Macclesfield, UK) were used to determine the Mw distribution of the AX in the samples with a flow rate of 0.6 mL/min for the mobile phase. 

##### 2.3.5.1. Construction of Standard Curve

Eight pullulan standards, as shown in Table 2, with an Mw range of 5000–800,000 Da were used to construct a standard curve for the determination of the molecular weight of AXs. The standard samples were dissolved into the mobile phase to make a 0.5 mg/mL solution and left overnight under gentle stirring. They were then filtered through a 0.45 μm nylon membrane before being transferred to a 1 mL glass shell vial.

**Table 2:** Molecular weight of pullulan standards.

Samples	Molecular weight (Da)
P-800	708,000
P-400	375,000
P-200	200,000
P-100	107,000
P-50	47,100
P-20	21,100
P-10	11,000
P-5	5900

##### 2.3.5.2. Determination of AXs Samples

The ethanol purified AX sample was dissolved in the solution of the mobile phase to make a 2 mg/mL solution and left overnight under gentle stirring. Then, it was filtered through a 0.45 μm nylon membrane and transferred to a 1 mL glass shell vial. The mobile phase for SEC-HPLC analysis was prepared by dissolving 17 g NaNO_3_ and 0.65 g NaN_3_ in HPLC-grade water into a 2000 mL flask to make up the mobile phase buffer. 

Each sample (100 µL) was injected and eluted at a flow rate of 0.6 mL/min and at ambient temperature. The column was calibrated, and the response factors were determined by injecting a solution mixture of eight pullulan standards. The Mw of the AX samples was identified by comparing their relative retention times with the standards. All analyses were conducted in duplicates, and the values were required to be within a 5% coefficient of variation. 

## 3. Results and Discussion

### 3.1. Chemical Composition of AEM

**Figure 1:**
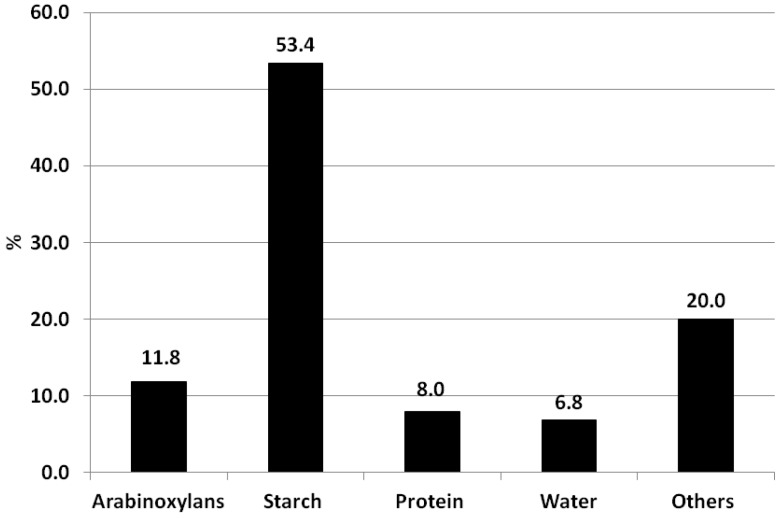
Chemical composition of arabinoxylan-enriched material provided by Henan Lianhua Monosodium Glutamate Group Co. Ltd.

The AEM from Henan Lianhua Monosodium Glutamate Group Co. Ltd. was separated from wheat flour through a high pressure disintegration processing production. The chemical composition of AEM was analysed (Figure 1). The results show that AEM contains 53.4% of starch, 8% of proteins and 11.8% of wheat endosperm AXs. This illustrates that the AX content of AEM was higher than that of untreated wheat grain (about 2%–5 % AXs) [6].

Even though the previous work has shown the strong potential to isolate AXs from wheat bran to produce the AX-enriched material with various methods [19,20], it was not feasible for industrial production, due to the high extraction cost. Wheat flour contains 1.5%–2.5% of AXs [21]. Generally, 20%–30% of AXs can be extracted by water (WE-AX); the remaining AXs are water unextractable (WU-AX). Wheat endosperm AXs have been isolated directly from wheat flour by removing starch and protein with enzymes in previous research [22,23]; however, there is no commercial wheat endosperm AX available as a food ingredient. The AEM in the current study has the potential to be marketed as a commercial wheat endosperm AX source.

### 3.2. Incorporation of AEM into Bread

**Figure 2:**
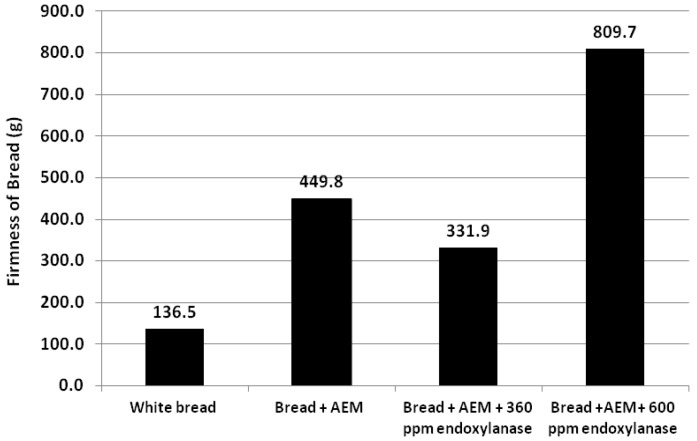
Bread crumb firmness of white bread, bread + AEM, bread + AEM with 360 ppm of endoxylanase and bread + AEM with 600 ppm of endoxylanase.

**Figure 3:**
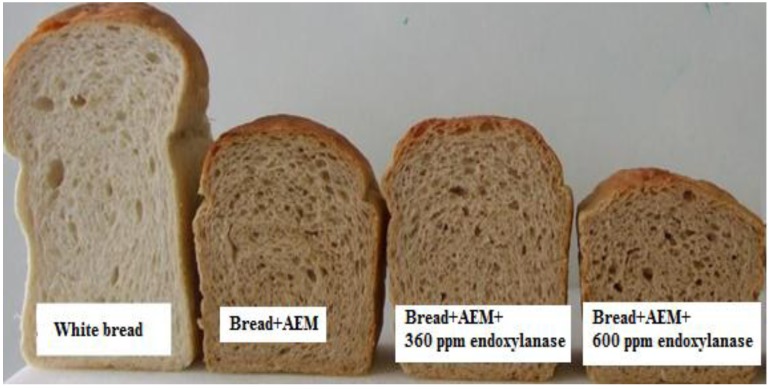
Bread crumb of white bread, bread + AEM, bread + AEM with 360 ppm of endoxylanase and bread + AEM with 600 ppm of endoxylanase.

AEM was incorporated into the bread as a source of wheat endosperm AXs (bread + AEM) for the development of functional food aiming for the modulation of post-prandial glycaemic response. As a result of such addition, the AX content increased to 6 g/100 g carbohydrate (based on 70% starch in flour). However, the incorporation of AEM exhibited strong negative impact on the bread quality in terms of bread crumb firmness and bread volume (Figure 2, Figure 3, respectively). The results show that the volume of the bread was significantly reduced and that this was associated with an increase in bread crumb firmness compared to the control white bread sample.

**Figure 4:**
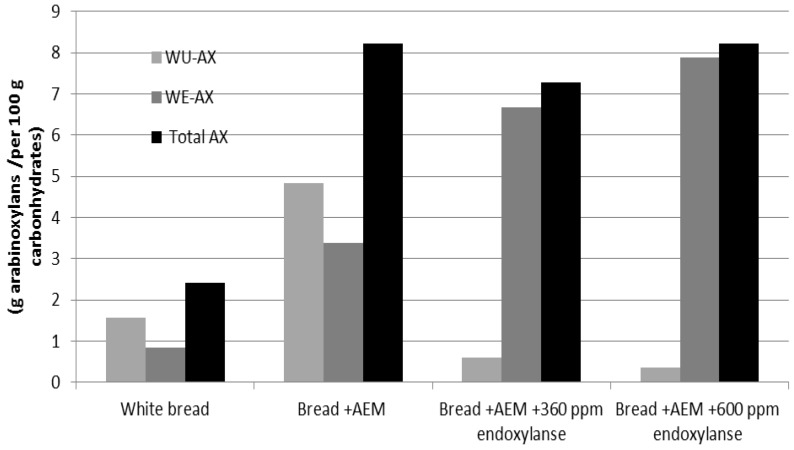
Water unextractable arabinoxylan (WU-AX), water extractable arabinoxylan (WE-AX) and total arabinoxylan content per 100 g of available carbohydrates in white bread, bread + AEM, bread + AEM with 360 ppm of endoxylanase and bread + AEM with 600 ppm of endoxylanase.

The functionality of wheat flour AXs in bread making has been investigated by Courtin and Delcour [24], Cleemput *et al.* [25] and Goesaert *et al*. [26]. These previous studies have shown that the WE-AX of wheat flour may have a positive effect on bread characteristics (texture and volume) and that those effects varied with the changes of their Mw [24,27]. It has also been illustrated that the WU-AX content of a product was negatively correlated with bread dough characteristics [27]. In the current study, the incorporation of AEM demonstrated significant negative effects on bread characteristics in terms of the bread volume and firmness, which may be attributed to the WU-AX of the AEM added (see Figure 4).

The commercial endoxylanase (Pentopan Mono BG) used in the current study is a purified 1,4-beta-xylanase (pentosanase) from *Thermomyces lanuginosus* (donor)/*Aspergillus oryzae* (host). The enzyme belongs to the family 11 of glycosyl hydrolases [28]. It is able to cleave the glycosidic bonds in the xylan backbone, bringing about a reduction in the degree of polymerization of the substrate. However, xylan is not attacked randomly, but the bonds selected for hydrolysis depend on the nature of the substrate molecule, *i.e.*, on the chain length, the degree of branching and the presence of substituents [29,30]. So far, it has been widely used in the baking industry with a recommended 10–30 ppm, based on flour weight.

The sensitivity of endoxylanase on arabinoxylan molecules has been widely investigated by Courtin *et al.* [26], Vardakou *et al.* [31] and Pitkanen *et al.* [32]. It has been well documented that inclusion of endoxylanase was able to increase both loaf volume and improve crumb structure by breaking down AXs [20,33] and converting WU-AX to WE-AX in bread dough [24]. In the current study, as the incorporation of AEM increased the AX content in flour, higher dosages of endoxylanase were selected for bread + AX + endoxylanase (E) (360 and 600 ppm) for a significant Mw depolymerisation of arabinoxylans [34].

Bread volume of the AEX samples increased with the addition of 360 ppm commercial endoxylanase (Figure 3), and crumb firmness decreased (Figure 2). The results demonstrated that added endoxylanase at this level diminished the negative effects of WU-AX on the bread + AEM. This is most likely due to the conversion of WU-AX to WE-AX in bread dough with the presence of 360 ppm of endoxylanase. Such an observation is consistent with the study of Damen *et al.* [7], where the added endoxylanase resulted in a good quality of rye and wheat bran fortified bread by degrading the arabinoxylans. The similar degradability of insoluble arabinoxylans was also observed by Arnaut *et al.* [34] in bread and Makaravicius *et al.* [35] in wheat and rye wholemeal treated by extrusion.

In contrast, the addition of 600 ppm of endoxylanase caused a further decrease in the volume of bread and a significant increase in the bread crumb firmness compared to the bread + AEM. In fact, the addition of 600 ppm endoxylanase enlarged the adverse effects of the WU-AX of the AEM on the bread. It is obvious that in the presence of 600 ppm of endoxylanase, the conversion of WU-AX to WE-AX would not be a dominating reaction to affect the quality of bread + AEM; The following investigation on the changes of extractability and the Mw distribution of AX in those breads may provide a better explanation.

### 3.3. Extractability of AXs in Bread

The WE-AX was extracted from tested bread and the AEM material. The content of the WE-AX and WU-AX of the AEM and the breads in the presence and absence of endoxylanase were determined by following the Douglas’s method [18]. The results (Figure 4) show that white bread contains 2.42 g of AX per 100 g available carbohydrates (calculated based on 75% starch of dry bread weight). This AX fraction contains 65% WU-AX and 35% WE-AX. As AEM was incorporated into the bread, the total AX of bread + AEM increased to 8.2 g per 100 g available carbohydrates (4.8 g of WU-AX and 3.4 g of WE-AX). In the bread + AEM with the presence of 360 ppm of endoxylanase, WE-AX increased to 6.6 g per 100 g available carbohydrates, but the WU-AX significantly decreased to 0.60 g per 100 available carbohydrates. With the presence of 600 ppm of endoxylanase, WE-AX increased further to 7.8 g per 100 g available carbohydrates with 0.43 g of WU-AX. 

The incorporation of AEM increased the WU-AX to 4.8 g per 100 g available carbohydrates, which may be the key factor for the strong negative effects on the bread quality of bread + AEM (Figure 2， Figure 3). In the presence of 360 ppm endoxylanase in bread + AEM, WU-AX was reduced from 4.8 g to 0.60 g as the WE-AX increased to 6.6 g per 100 g carbohydrates. Clearly, the significant increase in WE-AX with the decrease of WU-AX in bread + AEM has a positive effect on the characteristics of bread. With the addition of 600 ppm endoxylanase, the WE-AX further increased (7.8 g) and WU-AX decreased (0.36 g) compared with bread + AEM in the presence of 360 ppm endoxylanase. However, it showed strong negative effects on bread physical characteristics, suggesting that those negative effects may be related to the changes in the molecular weight.

### 3.4. Characterization of Mw of the WE-AX of the AEM and Tested Breads

**Figure 5:**
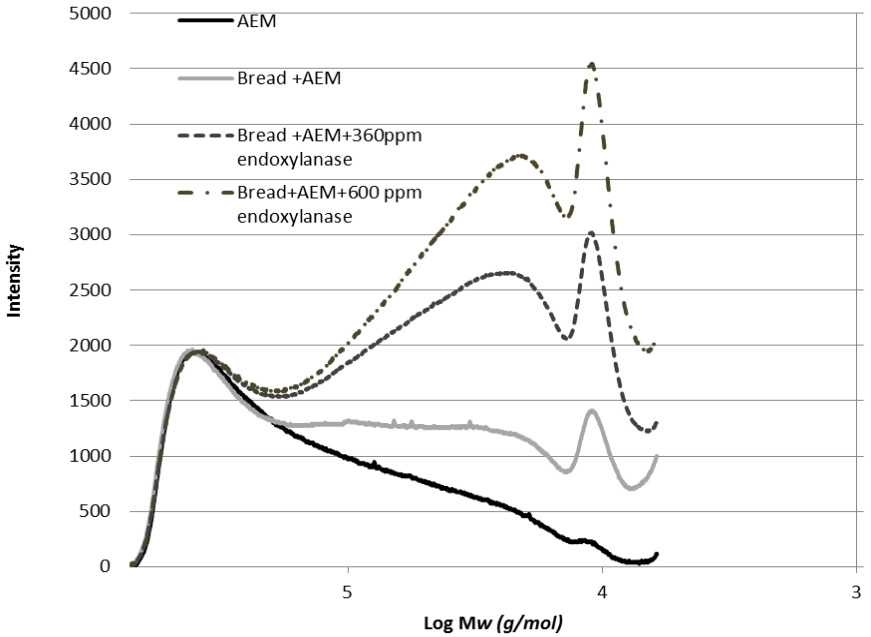
Molecular distribution of AX in AEM, bread + AEM, bread + AEM with 360 ppm of endoxylanase and bread + AEM with 600 ppm of endoxylanase.

The Mw distribution of the AXs of the AEM and the WE-AX of breads was characterized by SEC-HPLC. As the results shown in Figure 5, the WE-AX Mw of AEM was distributed in the range of 6–600 kDa, in which there was about 67% of WE-AXs with an Mw higher than 100 kDa, and 33% of WE-AXs have an Mw lower than 100 kDa. As AEM was incorporated into the bread, the portion of WE-AX with an Mw lower than 100 kDa increased from 33% to 42%. This indicates that in bread making, the Mw of WE-AX in the AEM shifted from the higher Mw to the lower Mw range, which might be due to depolymerisation, as a consequence of the mixing of bread dough and/or hydrolysis of the endoxylanase of wheat flour. As 360 ppm and 600 ppm of endoxylanase was added into the bread + AEM, the portion of WE-AX with an Mw lower than 100 kDa increased from 42% to 75% and further to 85%, respectively. Consequently, there were 5 g and 6.7 g of WE-AX with an Mw lower than 100 kDa per 100 g available carbohydrates for the bread, corresponding to the addition of 360 ppm and 600 ppm arabinoxylanase, respectively. The higher portion of WE-AX with the lower Mw may have a negative effect on the quality of the bread + AEM in the presence of 600 ppm arabinoxylanase.

Previous research has reported that wheat endosperm AXs have a functionality of regulating blood glucose levels [8,9,36]. When the AEM was incorporated into bread, the total AX was increased to 8.2 g per 100 g available carbohydrates. This level meets the requirement of EFSA regulation [37] for health claims of reduction of post-prandial glycaemic response. Hence, AEM is a good source of wheat endosperm AX for adding health benefits to food products. Based on the findings of the stronger immune stimulating properties of AXs with an average Mw of 32.5 kDa, rather than with an average Mw of 351 kDa [10,11], it can be postulated that the increase in the portion of AXs with an Mw lower than 100 kDa in bread + AEM with the presence of 360 ppm endoxylanase may be linked with the potential improvement of the immune stimulating activity of WE-AX.

Additionally, it has been noted that the Mw of AXs was an important parameter for prebiotic effects. Hughes *et al.* [38] discovered more pronounced effects for lower-molecular-weight (66 kDa) AXs in an *in vitro* study, which, in turn, led to the observation that prebiotic indices of enzyme-treated wheat AXs were higher than those of untreated AXs [39]. More interestingly, as wheat bran arabinoxylan was depolymerised to the AX oligosaccharides (AXOS) with a certain average DP of 15 and with an average degree of arabinose substitute of 0.26, stronger probiotic effects were observed [7,12,13,14,15]. In the bread + AEM and with the presence of 360 ppm and 600 ppm of AXs, the significant increase in the portion of AX with an Mw in the range of 1–10 kDa may result in the enhancement of prebiotic activity of the WE-AX in those kinds of bread. 

It is clear that the molecular characteristics of AXs need to be modified and to be consistent for achieving optimum immune stimulation, anti-tumour and prebiotic activities. In the current study, the Mw of AX in bread + AEM was modulated by the addition of endoxylanase. The significant increase in WE-AX in bread + AEM and bread + AEM with the presence of moderate amount of endoxylanase may help to reduce the post-prandial glycaemic response. The further molecule breakdown may result in the improvement of the immune stimulation properties and prebiotic activity of AXs. 

## 4. Conclusions

The study results show that the AEM separated from wheat flour, containing 11.8% AXs, is a good source of wheat endosperm AXs. It is achievable to incorporate 8.2 g of AX per 100 g of available carbohydrates into bread for the health claim of the reduction of the post-prandial glycaemic response. 

The presence of 360 ppm of endoxylanase significantly improved the firmness and volume of bread + AEM by converting WU-AX to WE-AX. In bread + AEM, with the presence of 360 ppm and 600 ppm of added endoxylanase, there was a significant increase in the portion of AXs with an Mw lower than 100 kDa in the bread.
